# The Effect of Dietary Polyphenols on Vascular Health and Hypertension: Current Evidence and Mechanisms of Action

**DOI:** 10.3390/nu14030545

**Published:** 2022-01-27

**Authors:** Giuseppe Grosso, Justyna Godos, Walter Currenti, Agnieszka Micek, Luca Falzone, Massimo Libra, Francesca Giampieri, Tamara Y. Forbes-Hernández, José L. Quiles, Maurizio Battino, Sandro La Vignera, Fabio Galvano

**Affiliations:** 1 Department of Biomedical and Biotechnological Sciences, University of Catania, 95123 Catania, Italy; giuseppe.grosso@unict.it (G.G.); currentiw@gmail.com (W.C.); m.libra@unict.it (M.L.); fgalvano@unict.it (F.G.); 2 Institute of Nursing and Midwifery, Faculty of Health Sciences, Medical College, Jagiellonian University, 31-501 Krakow, Poland; agnieszka.micek@uj.edu.pl; 3 Epidemiology Unit, IRCCS Istituto Nazionale Tumori “Fondazione G. Pascale”, 80131 Naples, Italy; l.falzone@istitutotumori.na.it; 4 Research Center for Prevention, Diagnosis and Treatment of Cancer, University of Catania, 95123 Catania, Italy; 5 Research Group on Food, Nutritional Biochemistry and Health, Universidad Europea del Atlántico, 39011 Santander, Spain; f.giampieri@univpm.it (F.G.); jlquiles@ugr.es (J.L.Q.); 6 Department of Physiology, Institute of Nutrition and Food Technology ‘‘José Mataix”, Biomedical Research Centre, University of Granada, 1800 Granada, Spain; tforbes@uvigo.es; 7 Department of Clinical Sciences, Polytechnic University of Marche, 60131 Ancona, Italy; m.a.battino@univpm.it; 8 International Joint Research Laboratory of Intelligent Agriculture and Agri-Products Processing, Jiangsu University, Zhenjiang 212013, China; 9 Department of Clinical and Experimental Medicine, University of Catania, 95131 Catania, Italy; sandrolavignera@unict.it

**Keywords:** polyphenols, flavonoids, phenolic acids, hypertension, blood pressure, endothelial

## Abstract

The aim of this review was to explore existing evidence from studies conducted on humans and summarize the mechanisms of action of dietary polyphenols on vascular health, blood pressure and hypertension. There is evidence that some polyphenol-rich foods, including berry fruits rich in anthocyanins, cocoa and green tea rich in flavan-3-ols, almonds and pistachios rich in hydroxycinnamic acids, and soy products rich in isoflavones, are able to improve blood pressure levels. A variety of mechanisms can elucidate the observed effects. Some limitations of the evidence, including variability of polyphenol content in plant-derived foods and human absorption, difficulty disentangling the effects of polyphenols from other dietary compounds, and discrepancy of doses between animal and human studies should be taken into account. While no single food counteracts hypertension, adopting a plant-based dietary pattern including a variety of polyphenol-rich foods is an advisable practice to improve blood pressure.

## 1. Introduction

Dietary risk factors represent a heavy burden for global chronic, non-communicable diseases [1]. There is consolidated evidence that dietary factors play a central role in determining cardiovascular disease (CVD) through a number of potential mechanisms. Obesity, type-2 diabetes, dyslipidemias, and hypertension are intermediary conditions representing key risk factors for CVD development, all strongly influenced by nutrition as well [2]. Among various potential effects on human health, dietary patterns such as the Mediterranean diet, the dietary approach to stop hypertension (DASH) model, Nordic diet, and lacto-ovo vegetarian have been demonstrated to play a role in preserving vascular health and reducing blood pressure [3]. A common feature of such dietary patterns is the richness in plant-derived foods, which are rich in fiber and phytochemicals with proven antioxidant activity, that have been studied over the last decades to explain the potential anti-hypertensive effects of the aforementioned diets.

**Figure 1:**
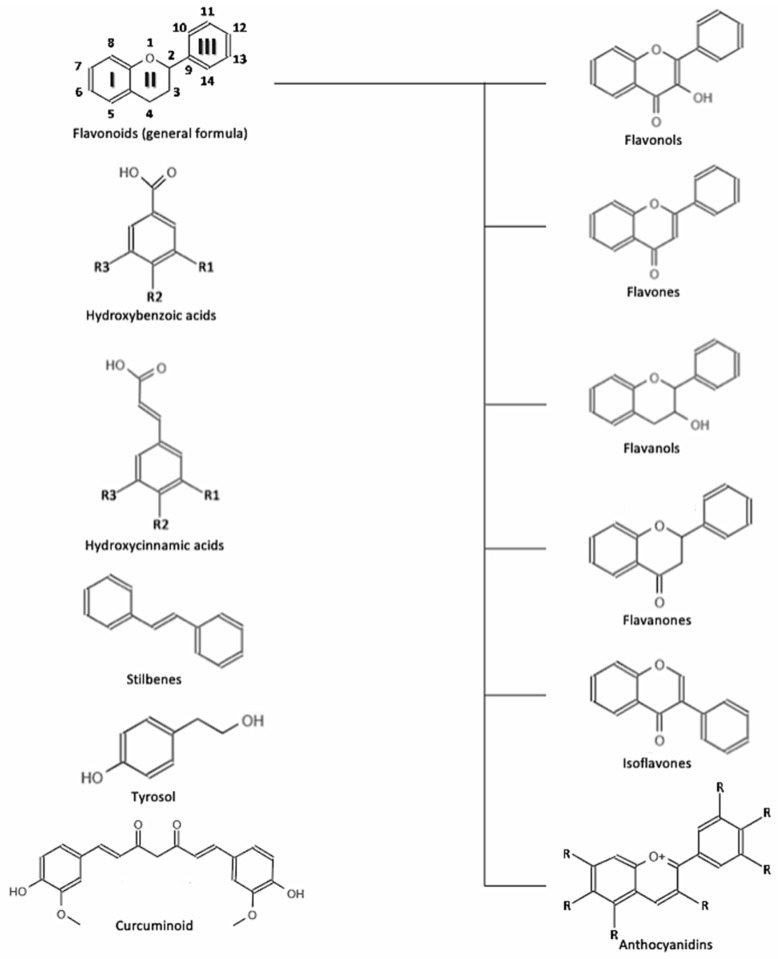
Chemical structure of the selected polyphenols.

Polyphenols are a large group of plant secondary metabolites that exert a number of biological activities involved in plant defense, including antioxidant and antibacterial actions [4]. While a large variety including thousands of molecules have been found in plants, some of them have also been proven to exert biological activities in humans [4]. The general chemical structure of plant polyphenols is characterized by one or more hydroxyl groups binding to one or more aromatic rings; besides being linked with one or more sugar residues, they can be associated also with other compounds, such as amines, carboxylic and organic acids, lipids and other phenols [5]. Based on their chemical structure (the number of phenol rings and structural elements that bind them) polyphenols are divided into groups and subgroups (Figure 1), mainly represented by flavonoids (further divided into flavonols, flavan-3-ols, anthocyanidins, flavones, flavanones, isoflavones, and chalcones) and “non-flavonoids” comprising phenolic acids, tyrosols, stilbenes, lignans, saponin, and tannins [5]. The structural diversity of these molecules affects their properties, thus yielding to different potential activities across different groups [4].

Polyphenols are found in different quantities and proportions in most edible and wild plants, fruits, and plant-derived beverages, but their consumption in humans is widely affected by the variety of the diet and, specifically, of plant-derived foods. In fact, consuming a high proportion of fruits and vegetables generally leads to a high dietary content of flavonoids [6]; populations consuming a high quantity of tea and coffee (i.e., northern and eastern European countries) have high dietary content of phenolic acids (i.e., caffeic acid) and catechins [7]; moreover, populations commonly using extra-virgin olive oil as dressing or red wine as alcohol during meals (i.e., Mediterranean countries) have a characteristic high intake of tyrosols and stilbenes [8]. Moreover, the bioavailability of polyphenols is generally low, with most of the compounds poorly absorbed in the small intestine, reaching the colon, where they are transformed by the colonic flora and later absorbed [9]. Importantly, the relation between polyphenols and gut microbiota is bidirectional, since the amount and types of molecules ingested have been shown to modulate the human gut microbiome community [10,11]. Thus, the overall absorption and, consequently, the potential effects of polyphenols may vary greatly depending on the content and variety of the overall diet (i.e., content in fiber, richness and variety of fruits and vegetables, consumption of coffee and tea, etc.).

In the most recent meta-analysis on the relation between dietary flavonoid consumption and cardiovascular outcomes [12] conducted on 39 prospective cohort studies including about a million and a half individuals, we reported that increasing consumption of flavonoids was linearly associated with a lower risk of CVD. Moreover, several other associations were found among main subclasses, including the linear association between anthocyanin and flavan-3-ol intake and CVD risk, flavonol and flavone intake and CHD risk, and flavanone intake and stroke risk [12]. Given the linear dose–response relation between flavonoid consumption and CVD risk, these results provide strong evidence of their effects on cardiovascular health. These potential protective effects are, at least in part, driven by their actions on vascular health and blood pressure regulation. There is plenty of literature describing putative mechanisms providing the rationale for a direct effect of dietary polyphenol intake on vascular health. The aim of this review is to summarize updated scientific literature published over the last few years on the relation between dietary polyphenols and polyphenol-rich foods on blood pressure, vascular endothelium health, and hypertension risk, as well as to elucidate the main mechanisms underlying the retrieved findings.

## 2. Evidence on Polyphenol and Hypertension

### 2.1. Observational Studies

We recently performed a systematic review and meta-analysis of observational studies on dietary polyphenol consumption and risk of hypertension including 15 cross-sectional investigations and 7 prospective cohorts [13]. The meta-analysis of five prospective cohorts, comprising 200,256 individuals and 45,732 cases of hypertension, included in the quantitative analysis showed that total flavonoids was not associated with the risk of hypertension, while among individual subgroups, anthocyanin intake was consistently associated with reduction in hypertension risk; among other observational studies reviewed, individuals consuming a higher intake of phenolic acids (such as hydroxycinnamic acids) [14,15] and phytoestrogens (including isoflavones) [16,17] were less likely to be hypertensive [13].

Concerning polyphenol-rich foods, a comprehensive summary of evidence from umbrella meta-reviews showed a decreased risk of hypertension associated with higher consumption of plant-based foods, including fruit, whole grains, nuts, and legumes/pulses [18,19,20,21,22], although some of the meta-analyses included were of relatively low quality, thus undermining the overall strength of the evidence. Null results were rather found for vegetable intake and risk of hypertension [23,24]. Concerning plant-derived beverages rich in polyphenols, we performed the most complete meta-analysis on long-term coffee consumption and risk of hypertension, including seven cohorts and 205,349 individuals and 44,120 cases of hypertension, which showed a linear dose–response association [25].

### 2.2. Dietary Intervention Trials

**Figure 2:**
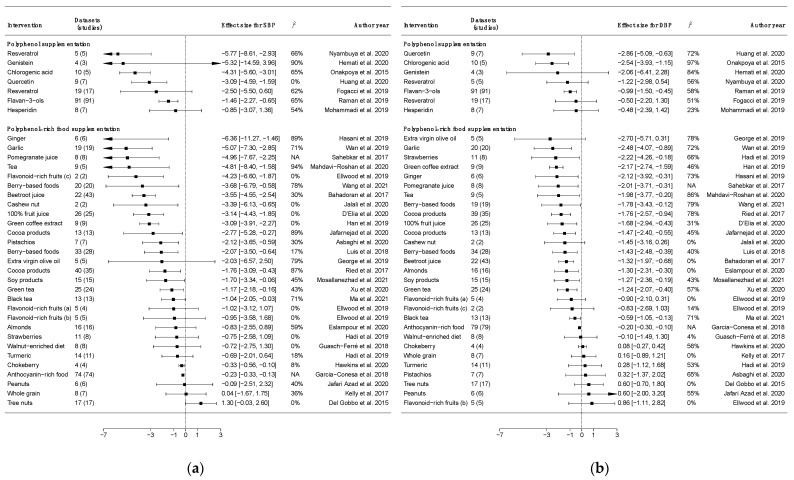
Summary of the main results from meta-analyses of randomized controlled trials (RCTs) investigating the effect of polyphenol and polyphenol-rich food supplementation on (**a**) systolic blood pressure (SBP), and (**b**) diastolic blood pressure (DBP). NA denotes not applicable.

A summary of the main results from meta-analyses of randomized controlled trials (RCTs) is presented in Figure 2.

Some studies investigating polyphenol supplementation consistently revealed null effects on blood pressure, suggesting that the lack of significant results may be due to the limited number of included studies and their quality; among the existing meta-analyses, supplementation with hesperidin (a major flavanone contained in citrus fruits) [26], genistein (an isoflavone contained in soy products) [27], and resveratrol (a stilbene contained in grapes and red wine) [28] led to null results on blood pressure outcomes. However, findings from other meta-analyses on resveratrol supplementation revealed an effect on flow-mediated dilatation (FMD) levels (1.77, 95% CI: 0.25 to 3.29, *p* = 0.02; I2: 96%) [29] and lowered systolic blood pressure (−5.77, 95% CI: −8.61 to −2.93) when considering diabetic patients [30]. A meta-analysis including RCTs supplementing patients with quercetin (another major flavonoid contained in onions and apples) showed a significant effect on both systolic (−3.09 mmHg, 95% CI: −4.59 to −1.59, *p* = 0.0001) and diastolic blood pressure (−2.86 mmHg, 95% CI: −5.09 to −0.63, *p* = 0.01) [31], despite the fact that no effects were detected on vascular cell adhesion molecule 1 (VCAM-1) of intercellular adhesion molecule 1 (ICAM-1) [32]. A meta-analysis of 91 RCTs comparing the effect of flavan-3-ols (flavonoids contained in cocoa and green tea) with controls on blood pressure showed a significant decrease in systolic (−1.46 mmHg, 95% CI: −2.27 to −0.65; I2 = 65.3%) and diastolic blood pressure (−0.99 mmHg, 95% CI: −1.50 to −0.45; I2 = 58.0%) [33]. Finally, a meta-analysis showed a significant systolic blood-pressure-lowering effect following chlorogenic acid supplementation (a major coffee polyphenol belonging to the hydroxycinnamic acid group) (−4.31 mmHg, 95% CI: −3.91 to −3.45, *p* < 0.001) [34]. However, the inclusion of a small number of participants and funding from manufacturers of original RCTs generally limited the strength of evidence due to possible bias.

Dietary intervention trials aiming to investigate the role of dietary polyphenols on blood pressure are typically characterized by supplementation with a polyphenol-rich food (or food group) in comparison to a control group. Regarding the potential effect of fruits on blood pressure, a recent meta-analysis on flavonoid-rich fruits (assessing the potential effect of anthocyanins, naringin, narirutin, and flavan-3-ols) including 15 RCTs and 572 participants showed no effect of flavonoids on systolic or diastolic blood pressure when compared to the placebo; however, pooled results from two crossover RCTs evidenced a reduction in systolic blood pressure [35]. Among specific fruits, a meta-analysis of 28 intervention studies on berry-based foods (rich in anthocyanins and some flavonols) showed an improvement in systolic (−2.07 mmHg, 95% CI: −3.50 to −0.64, *p* = 0.005) and diastolic blood pressure (−1.43 mmHg, 95% CI: −2.47 to −0.38, *p* = 0.007), while no effects were found on VCAM (−21.00, 95% CI: −58.75 to 16.74) and ICAM (−0.103, 95% CI: −0.481 to 0.275) [36]. Similarly, another meta-analysis conducted on anthocyanin-containing products (including berries, red grapes, and red wine) showed a decrease in blood pressure (systolic, −0.23, *p* < 0.001; diastolic, −0.20, *p* < 0.001) [37]. These results have been confirmed in a more recent meta-analysis on berries (including juice of barberry, cranberry, grape, pomegranate, powder of blueberry, grape, raspberry and freeze-dried strawberry), showing a significant reduction in systolic blood pressure by 3.68 mmHg (95% CI −6.79 to −0.58, *p* = 0.02) and diastolic blood pressure by −1.78 mmHg (95% CI −3.43 to −0.12, *p* = 0.04) and elevated sVCAM-1 levels by 14.57 ng/mL (85% CI 4.22 to 24.93; *p* = 0.02) [38]. Other meta-analyses focused on specific berry fruits reported an individual positive impact on systolic blood pressure with the consumption of strawberries [39] and chokeberry (aronia melanocarpa) [40]. Concerning fruit juices, a meta-analysis on 100% fruit juices showed a favorable effect on blood pressure (systolic, MD: −3.14 mmHg; diastolic, MD: −1.68 mmHg), arterial compliance (carotid–femoral pulse wave velocity, −0.38 m/s), and endothelial function (flow-mediated dilation, 2.10%) [41]. Among specific juices, a meta-analysis including eight RCTs on pomegranate juice found significant reductions in both systolic (−4.96 mmHg, 95% CI: −7.67 to −2.25, *p* < 0.001) and diastolic blood pressure (−2.01 mmHg, 95% CI: −3.71 to −0.31, *p* = 0.021) after pomegranate juice (rich in anthocyanin) consumption [42]. Another meta-analysis including 22 trials (1248 participants) on beetroot juice (rich in anthocyanin) showed a lower mean difference of systolic (−3.55 mmHg; 95% CI: −4.55 to −2.54) and diastolic blood pressure (−1.32 mmHg; 95% CI: −1.97 to −0.68) in the intervention compared to control groups [43].

Although no significant association between total vegetable intake and risk of hypertension has been found in observational studies, a meta-analysis of RCTs on garlic (rich in hydroxycinnamic acid derivatives) revealed lowering effects on systolic (−5.07 mmHg; 95% CI −7.30 to −2.85) and diastolic blood pressure (−2.48 mmHg; 95% CI −4.07 to −0.89) [44]. Supplementation with vegetables such as turmeric (rich in curcuminoids) has been reported not to exert significant effects on blood pressure, although a significant reduction in systolic (−1.24 mmHg, 95% CI: −2.26 to −0.22; I2 = 0%) but not diastolic blood pressure (0.29 mmHg, 95% CI: −0.65 to 1.22; I2 = 1%) has been observed when restricting the analysis on studies with a duration >12 weeks [45]. Among other plant-derived foods, a meta-analysis including eight RCTs (768 participants) on whole-grain supplementation (containing a variety of phenolic acids and lignans) showed no effects on either systolic (0.04 mmHg, 95% CI: −1.67 to 1.75) or diastolic blood pressure (0.16, 95% CI: −0.89 to 1.21) [46]. While no RCTs have been conducted on pulses, a meta-analysis on soy products (characterized by high content in isoflavones) including 15 RCTs showed a significant reduction in both systolic (−1.70 mmHg, 95% CI: −3.34 to −0.06, *p* = 0.04; I2 = 45%) and diastolic blood pressure (−1.27 mmHg, 95% CI: −2.36 to −0.19, *p* = 0.02; I2 = 43%) [47]. A meta-analysis on tree nuts (high in phenolic acids, including hydroxybenzoic acids) showed no direct effects on blood pressure levels following ingestion of nuts with no differentiation among types [48]. When considering specific nut types, walnut-enriched diets and peanut supplementation did not lead to significant differences in blood pressure levels [49,50]. However, a meta-analysis specifically conducted on almonds including 16 RCTs and 1128 participants showed a reduction in diastolic (−1.30 mmHg, 95% CI: −2.31 to −0.30; I2 = 0.0%) but not systolic blood pressure levels [51]. In contrast, in another meta-analysis on pistachio supplementation including 13 RCTs with 563 participants, a significant decrease in systolic blood pressure (−2.12 mmHg, 95% CI −3.65 to −0.59, *p* = 0.007) was found, whereas changes in flow-mediated dilation and diastolic blood pressure were not significant [52]. Similarly, in another meta-analysis on cashew nut supplementation, although limited to two RCTs and 123 participants, no effect was recorded for diastolic blood pressure, but a significant reduction in systolic blood pressure (−3.39 mmHg, 95% CI = −6.13 to −0.65; I2 = 0.0%) in the intervention compared to the control group was found [53].

Concerning cocoa products (rich in flavan-3-ols), a meta-analysis of 35 trials (40 treatment comparisons) involving 1804 mainly healthy participants showed a significant blood pressure-reducing effect of flavanol-rich cocoa products compared with the control in trials (−1.76 mmHg, 95% CI: −3.09 to −0.43, *p* = 0.009, and −1.76 mmHg, 95% CI: −2.57 to −0.94, *p* < 0.001, respectively) [54]. A meta-analysis including RCTs restricting the interventions in middle-aged and elderly individuals showed a significant reduction in systolic blood pressure by 2.77 (95% CI: −5.28 to −0.27, *p* = 0.03; I2 = 89%) and diastolic blood pressure by 1.47 mmHg (95% CI: −2.40 to −0.55, *p* = 0.001; I2 = 45%) [55]. Another meta-analysis including 15 RCTs with 18 intervention arms estimating a 704 mg/d higher intake of cocoa flavan-3-ols on average than the control revealed a significant improvement of FMD by 1.17% (95% CI: 0.76% to 1.57%) [56].

While no intervention trials are available for regular coffee consumption and blood pressure, a meta-analysis of nine RCTs on green coffee extract supplementation showed a significant reduction in systolic (−3.09 mmHg, 95% CI: −3.91 to −2.27; I2 = 0.0%) and diastolic blood pressure (−2.17 mmHg, 95% CI: −2.74 to −1.59; I2 = 46.5%) with low heterogeneity among the studies [57]. In contrast, a quantitative summary of evidence on tea consumption has been conducted including 13 trials (1115 participants) on black tea (rich in hydroxybenzoic acids) and 24 trials (1697 participants) on green tea supplementation (rich in flavan-3-ols, such as epigallocatechin-gallate) showing a significant reduction in both systolic (−1.04 mmHg, 95% CI: −2.05 to −0.03, *p* = 0.04, and −1.17 mmHg, 95% CI: −2.18 to −0.16, *p* = 0.02, respectively) and diastolic blood pressure (−0.59 mmHg, 95% CI: −1.05 to −0.13, *p* = 0.01, and −1.24 mmHg, 95% CI:−2.07 to −0.40, *p* = 0.004, respectively), although with some evidence of heterogeneity between studies [58,59]. An analysis restricted to studies conducted on individuals with high blood pressure (five RCTs on 408 participants) showed more clinically relevant effects on both systolic (−4.81 mmHg, 95% CI: −8.40 to −1.58, *p* = 0.004) and diastolic blood pressure (−1.98 mmHg, 95% CI: −3.77 to −0.20, *p* = 0.029) [60].

Among other foods rich in polyphenols, ginger is rich in gingerols and it has been studied for its potential effects on blood pressure; a meta-analysis including six RCTs and 345 participants showed that ginger supplementation would reduce both systolic (−6.36 mmHg, 95% CI: −11.27 to −1.46, *p* = 0.011; I2 = 89%) and diastolic blood pressure (−2.12 mmHg, 95% CI: −3.92 to −0.31, *p* = 0.002; I2 = 73%), although the overall level of evidence is relatively weak due to the high heterogeneity between studies and the small number of participants [61]. Additionally, olive oil is particularly rich in polyphenols, especially phenolic acids, which have been considered most probably responsible for the health benefits of this oil; however, a meta-analysis on high-polyphenol extra-virgin olive oil including five RCTs showed no direct effect on blood pressure levels (−2.03 mmHg, 95% CI: −6.57 to 2.50, *p* = 0.38; I2 = 79% for systolic blood pressure; −2.70 mmHg, 95% CI: −5.71 to 0.31, *p* = 0.08; I2 = 78% for diastolic blood pressure) [62].

## 3. Summary of Potential Mechanisms of Action

**Figure 3:**
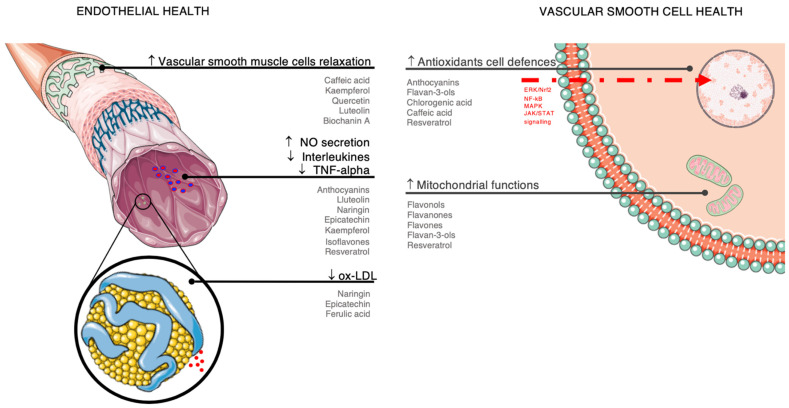
Summary of the potential mechanisms of action through which polyphenols may affect endothelial health and reduce risk of hypertension. ↑denotes increase, ↓denotes decrease.

A summary of the potential mechanisms of action through which polyphenols may affect endothelial health and reduce the risk of hypertension is shown in Figure 3.

### 3.1. Endothelial Health

Vascular functions, including vascular tone maintenance, redox balance, and inhibition of platelet aggregation and coagulation, are key factors for endothelial health and the prevention of hypertension, atherosclerosis, and CVD [63]. Endothelial cells produce substances needed for the maintenance of healthy vascular function, including nitric oxide (NO), carbon monoxide, endothelium-dependent hyperpolarizing factors and endothelium-derived contracting factors, vasoactive prostanoids and prostacyclin, endothelin, and superoxide [63]. Endothelial dysfunction is substantially driven by reduced availability of NO as a consequence of increased oxidative stress, generation of free radicals, and other stress factors; polyphenols may improve the release of NO from the endothelial cells, leading to activation of cyclic guanosine monophosphate in vascular smooth muscle cells and exerting blood vessel relaxation, antioxidant, anti-inflammatory, and antithrombotic effects [64]. Flavonoids, such as anthocyanins [65,66], flavones (i.e., luteolin) [67], flavanones (i.e., naringin) [68], flavan-3-ols (i.e., epicatechin) [69], flavonols (i.e., kaempferol) [70] and isoflavones [71], and resveratrol [72,73] may play a direct role in improving the bioavailability in the bloodstream of NO by increasing the activation of inducible NO synthase (iNOS) and endothelial NO synthase (eNOS) provided by modulation of signal transduction, for instance through the phosphatidylinositol 3-kinase (PI3K)/Akt or the adenosine monophosphate-activated protein kinase (AMPK) pathways [67]. Together with other polyphenols, such as caffeic acid [74], kaempferol [75], quercetin [76], luteolin [77], and biochanin A [78], these compounds may exert vasorelaxing effects also by acting on vascular smooth muscle cells directly (through activation of BK channels or inhibition of Ca^2+^ channels) or indirectly (through activation of Ca^2+^-activated K^+^ channels in endothelial cells, leading to hyperpolarization and inhibition of Ca^2+^ influx to vascular smooth muscle cells), eventually limiting construction and leading to vasorelaxation [79]. However, some polyphenols, such as resveratrol, have been shown to act through more than one of the aforementioned mechanisms [80].

### 3.2. Antioxidant Effects

Oxidative stress following free radical and reactive oxygen species (ROS) production is a cornerstone process in aging and disease, including atherosclerosis and CVD [81]. Regarding atherosclerosis development, oxidative modification of low-density lipoprotein (LDL) into oxidized-LDL (ox-LDL) represents one of the earliest events of the whole process; dietary polyphenols have been shown to counteract this process and exert antioxidant activity through a variety of endogenous and exogenous mechanisms [82]. The most common mechanism of action of flavonoids [83], including citrus flavonoids [84] and flavan-3-ols (i.e., epicatechin) [85], and phenolic acids, such as ferulic acid [86], against ROS have been shown to be the potent direct scavenging free radical activity. Flavonoids are oxidized by free radicals, resulting in more stable and less reactive compounds; among the most potent antioxidant compounds, flavonols (i.e., quercetin) [87], flavanones (i.e., naringenin and hesperetin), flavones (i.e., apigenin) [67], flavan-3-ols (i.e., catechins), stilbenes (i.e., resveratrol) [88], and many others have been shown to directly scavenge superoxide or other reactive species, reversing vascular stiffening and restoring its functionality. Polyphenols may provide antioxidant effects, counteracting senescence and restoring mitochondrial function vascular smooth muscle cells and endothelial cells by modulation of signal transduction [4]. For instance, flavonoid subgroups such as flavanones and anthocyanins, as well as phenolic acids such as caffeic acid, demonstrated antioxidant activity by enhancing the cellular antioxidant defenses through activation of transcription factors of antioxidant and cytoprotective enzymes, such as the extracellular signal-regulated kinase (ERK)/nuclear factor (erythroid-derived 2)-like 2 (Nrf2) signaling pathway [89,90]. Modulation of signal transduction may lead to the promotion of upregulation of anti-oxidative genes, such as heme oxygenase-1 (HO-1), NAD(P)H dehydrogenase quinone 1 (NQO1), glutamate–cysteine ligase (through its catalytic subunit–GCLC), and induction of endogenous antioxidant enzymes, such as glutathione peroxidase, superoxide dismutase, catalase, or glutathione reductase [91].

### 3.3. Anti-Inflammatory Action

Inflammation plays a central role in numerous non-communicable diseases, including cardiovascular disorders [92]. Concerning vascular health, migration and accumulation of ox-LDL cholesterol in the vascular intima has been long considered the main event for the determination of atheroschlerotic disease, while this interpretation of events is rather limited; in fact, it only represents the first step in the development of the disease, which also involves macrophages’ activity in discharging various mediators of inflammation, sustaining the whole process of infiltration of smooth muscle cells, formation of foam cells, and leukocytes infiltration/proliferation [93]. Inflammatory biomarkers characterizing the process include various cytokines (interleukin- (IL-) 1, 3, 6, 8, and 18), tumor necrosis factor (TNF-alpha), and the macrophage colony-stimulating factor; among polyphenols, some flavonoids such as anthocyanins [94] and flavan-3-ols [94], phenolic acids (including chlorogenic and caffeic acids) [95], and resveratrol [96] are promising anti-inflammatory agents due to their anti-inflammatory effects specifically interfering with the aforementioned mechanisms through regulation of several signaling pathways, including the mitogen-activated protein kinases (MAPK), the janus kinase/signal transducers/activators of the transcription (JAK/STAT), and the NF-κB pathways [97].

### 3.4. Platelet Adhesion, Aggregation, and Coagulation

Proinflammatory stimuli lead to changes in the endothelial phenotype, leading to damage of barrier function and upregulation of adhesion molecules expression, including vascular cell adhesion molecule-1 (VCAM-1) and intercellular adhesion molecule (ICAM-1) [98]. Such changes represent the final step toward plaque formation, necrotization, and rupture, with the establishment of atherosclerosis. Several polyphenol groups, such as apigenin [99], curcumin and luteolin [100,101], quercetin [102], epicatechin [103], and resveratrol [104], have been shown to reduce platelet aggregation through the aforementioned anti-inflammatory pathways and inhibition of adhesion molecules [105].

### 3.5. Potential Role of Gut Microbiota

Dietary polyphenols have been described to be of great relevance for the composition of the colonic bacterial flora [106]. Although heterogeneity in individuals’ characteristics (i.e., age, sex, ethnicity, BMI) causes large inter-individual variations in gut microbiota to occur, the bacterial production of molecules reaching the systemic circulation has been hypothesized to play an indirect role in vascular endothelial health [107]. Among the most studied compounds produced by the gut microbiota, the metabolite trimethylamine-N-oxide (TMAO), generated in the liver via the oxidation of trimethylamine (TMA) formed by *Firmicutes* and *Proteobacteria* from carnitine and choline-rich foods (i.e., red meat, dairy, eggs, poultry), has been demonstrated to induce vascular inflammation through mitogen-activated protein kinase (MAPK) and NF-κB signaling [108]. Polyphenols, such as resveratrol, have been shown to decrease circulating TMAO by regulating its synthesis via gut microbiota remodeling [109], with similar results confirmed in a recent RCT conducted on humans [110]. Additionally, tea flavonoids (i.e., flavan-3-ols) have been shown to alleviate atherosclerosis by decreasing serum TMA by regulating gut microbiota [111]. Other important gut microbiota-derived metabolites shown to be beneficial for blood vessel control are short-chain fatty acids (SCFAs), which are the result of bacterial (mainly Lactobacillus and Bifidobacterium) fermentation of undigested carbohydrates, which can cross the intestinal epithelium and influence mucosal immune responses [112]. In vivo studies showed that polyphenols, such as isoflavones [113], anthocyanins [114], and flavan-3-ols [115], have been shown to affect gut microbiota composition and increase fecal SCFA.

## 4. Limitation of the Evidence

Existing evidence from meta-analyses is generally limited by common general limitations, such as a small number of studies or participants, involvement of manufacturers, and scarce clinical relevance of some significant results (not enough variation in blood pressure). Moreover, while there is a certain agreement between studies conducted on humans and mechanistic studies from the laboratory setting, some limitations of the retrieved evidence may stem from the following: (i) large variability of polyphenol content in plant-derived foods (depending on cultivar, preservation, temperature, sun exposure, and other factors) as well as in absorption of polyphenols among humans; (ii) dietary sources of polyphenols are typically rich in other compounds that may exert cardioprotective effects, including fiber or monounsaturated fatty acids; thus, disentangling the unique effects of polyphenols is rather difficult when studying food groups; (iii) mechanistic studies conducted on cells and animals often do not take into account the cooking processes, which may lead to a loss in total polyphenol or transformation of specific molecules into others with different biological activities; and (iv) discrepancy between in vitro and in vivo doses (in vitro doses often lead to unrealistic dietary intake in humans).

## 5. Conclusions

In conclusion, evidence from human studies suggests that some polyphenol-rich foods exert positive effects on blood pressure levels. However, given the small clinical effects reported, the real-world implications for their consumption rely on their inclusion in a healthy diet rather than consumption of an individual food. There is no singular mechanism nor individual polyphenol compound that may explain the pathway to improve endothelial health and prevent hypertension and CVD. On the contrary, it is likely that the health benefits of plant-based diets rich in polyphenols may depend both on the quantity and the variety of compounds acting through several pathways, leading to synergistic actions toward health. Furthermore, a recommendation for the adherence to a healthy diet rich in plant-based foods, providing not only substrates to the gut microbiome (i.e., fiber) and important co-factors playing a role in polyphenol bioavailability but also influencing gut microbiota profile, could strengthen the beneficial effects of polyphenols toward cardiovascular health. Although recommendations on the consumption of single foods are inappropriate, it can be concluded that a diet rich in multiple polyphenol-rich foods is likely to improve vascular health and reduce the risk of hypertension.
